# Supplementary material for: Understanding early HIV-1 rebound dynamics following antiretroviral therapy interruption: The importance of effector cell expansion
Source: bioRxiv. 2024 May 5:2024.05.03.592318. Preprint. [Version 1] doi: 10.1101/2024.05.03.592318 (PMC11092759; doi:10.1101/2024.05.03.592318)
Supplement: 1 [file NIHPP2024.05.03.592318v1-supplement-1.pdf]

bioRxiv preprint doi: https://doi.org/10.1101/2024.05.03.592318; this version posted May 5, 2024. The copyright holder for this preprint (which was not certified by peer review) is the author/funder. This article is a US Government work. It is not subject to copyright under 17 USC 105 and is also made available for use under a CC0 license.

Supplementary Material

S1. Equations for each model

Here, we present the equations for all six models that we fit to the viral load data for the 24 PWH that we studied in the main text. Note that $V = \frac{p}{c} I$ in all models.

Conway & Perelson model

$$\begin{aligned} T' &= \lambda_T - d_T T - \beta V T \\ I' &= (1 - f_L)\beta V T + aL - \delta I - mEI \\ L' &= f_L \beta V T - d_L L - aL + \rho L \\ E' &= \lambda_E + \frac{bEI}{K_B + I} - \frac{dEI}{K_D + I} - d_E E. \end{aligned}$$

Simplified Model 1 (as above, but $d = 0$)

$$\begin{aligned} T' &= \lambda_T - d_T T - \beta V T \\ I' &= (1 - f_L)\beta V T + aL - \delta I - mEI \\ L' &= f_L \beta V T - d_L L - aL + \rho L \\ E' &= \lambda_E + \frac{bEI}{K_B + I} - d_E E. \end{aligned}$$

Simplified Model 2 (as Simplified Model 1, but no dynamics of latent cells $L' \equiv 0$)

$$\begin{aligned} T' &= \lambda_T - d_T T - \beta V T \\ I' &= (1 - f_L)\beta V T + aL_0 - \delta I - mEI \\ E' &= \lambda_E + \frac{bEI}{K_B + I} - d_E E. \end{aligned}$$

Simplified Model 3 (as Simplified Model 2, but $d \neq 0$, so with exhaustion of effector cells)

$$\begin{aligned} T' &= \lambda_T - d_T T - \beta V T \\ I' &= (1 - f_L)\beta V T + aL_0 - \delta I - mEI \\ E' &= \lambda_E + \frac{bEI}{K_B + I} - \frac{dEI}{K_D + I} - d_E E. \end{aligned}$$

Simplified Model 4 (as Simplified Model 2, but no explicit dynamics of effector cells $E' \equiv 0$)

$$\begin{aligned} T' &= \lambda_T - d_T T - \beta V T \\ I' &= (1 - f_L)\beta V T + aL_0 - \delta I - m_f I. \end{aligned}$$

Simplified Model 5 (as Simplified Model 1, but no explicit dynamics of effector cells $E' \equiv 0$)

$$\begin{aligned} T' &= \lambda_T - d_T T - \beta V T \\ I' &= (1 - f_L)\beta V T + aL - \delta I - m_f I \\ L' &= f_L \beta V T - d_L L - aL + \rho L. \end{aligned}$$

bioRxiv preprint doi: https://doi.org/10.1101/2024.05.03.592318; this version posted May 5, 2024. The copyright holder for this preprint (which was not certified by peer review) is the author/funder. This article is a US Government work. It is not subject to copyright under 17 USC 105 and is also made available for use under a CC0 license.

S2. Model fits

Table S1 shows the comparisons of models under different assumptions. Figures S1-S6 (and Figure 1, main text) are the best fit of each model, with the population estimates provided in Tables S2-S6. Figures S7-S10 show the stratifications of best fit parameters for the Conway & Perelson model, Simplified Models 1, 2 and 3. Tables S7-13 give the individual estimates for the best fit of the Conway & Perelson model and Simplified Models 1 (with and without covariate on K_B) through 5.

Model	Effector cell exhaustion	Dynamic latent reservoir	Dynamic effector cells	-2LL	BICc	Figure (Best-fit)
Conway & Perelson	Yes	Yes	Yes	547.38	625.29	S1
Simplified Model 1	No	Yes	Yes	**545.33**	**605.22**	1 (main)
Simplified Model 2	No	No	Yes	548.12	608.02	S3
Simplified Model 3	Yes	No	Yes	545.66	623.57	S4
Simplified Model 4		No	No	894.15	927.01	S5
Simplified Model 5		Yes	No	893.17	926.04	S6
Simplified Model 1 (covariate on K_B)				**515.74**	**578.81**	S2
Simplified Model 1 (covariate on β)				547.94	611.01	—
Simplified Model 1 (covariate on λ_E)				546.29	609.36	—
Simplified Model 1 (covariate on b)				532.59	595.66	—
Simplified Model 1 (covariate on p)				546.98	610.05	—
Simplified Model 1 (covariate on m)				543.33	606.40	—
Simplified Model 1 (covariates on K_B and m)				**513.58**	**579.83**	**—**
Simplified Model 1 (covariates on K_B and b)				514.84	581.09	—
Simplified Model 1 (without random effect for K_B)				593.83	650.55	—

Table S1. Model fit comparison (Monolix 2023R1, Lixoft, SA, Antony, France). There are four categories separated by double-lines. Bolded values are the lowest values in the category. The first category contains the Conway and Perelson model with five variations without covariates. The second category tests single covariates for the best fit model in the first category. The third category shows two examples of using two covariates that produce the lowest fitting error. The last category tests the Simplified Model 1 without random effect for K_B. Without a random effect, the population estimate for K_B is 16.88 (S.E. 0.12) cells mL^{-1}.

bioRxiv preprint doi: https://doi.org/10.1101/2024.05.03.592318; this version posted May 5, 2024. The copyright holder for this preprint (which was not certified by peer review) is the author/funder. This article is a US Government work. It is not subject to copyright under 17 USC 105 and is also made available for use under a CC0 license.

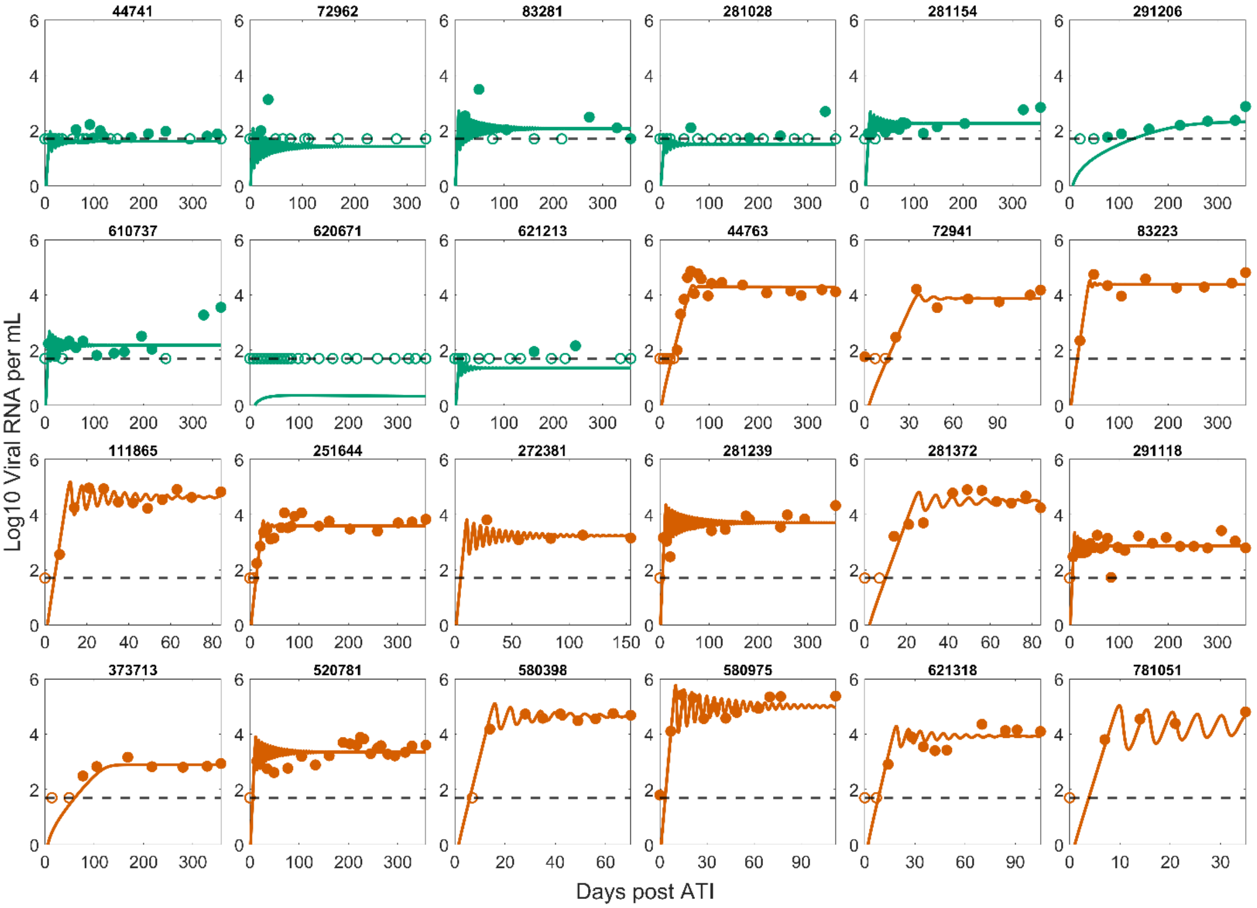

Figure S1. Best fit of the Conway & Perelson model to the post-ATI data from Sharaf et al. [57]. Green indicates PTC. Dark orange indicates NC. The horizontal dashed line is the limit of detection. Open circles are data points below the limit of detection (50 viral RNA copies/mL). Filled circles are data points above the limit of detection.

bioRxiv preprint doi: https://doi.org/10.1101/2024.05.03.592318; this version posted May 5, 2024. The copyright holder for this preprint (which was not certified by peer review) is the author/funder. This article is a US Government work. It is not subject to copyright under 17 USC 105 and is also made available for use under a CC0 license.

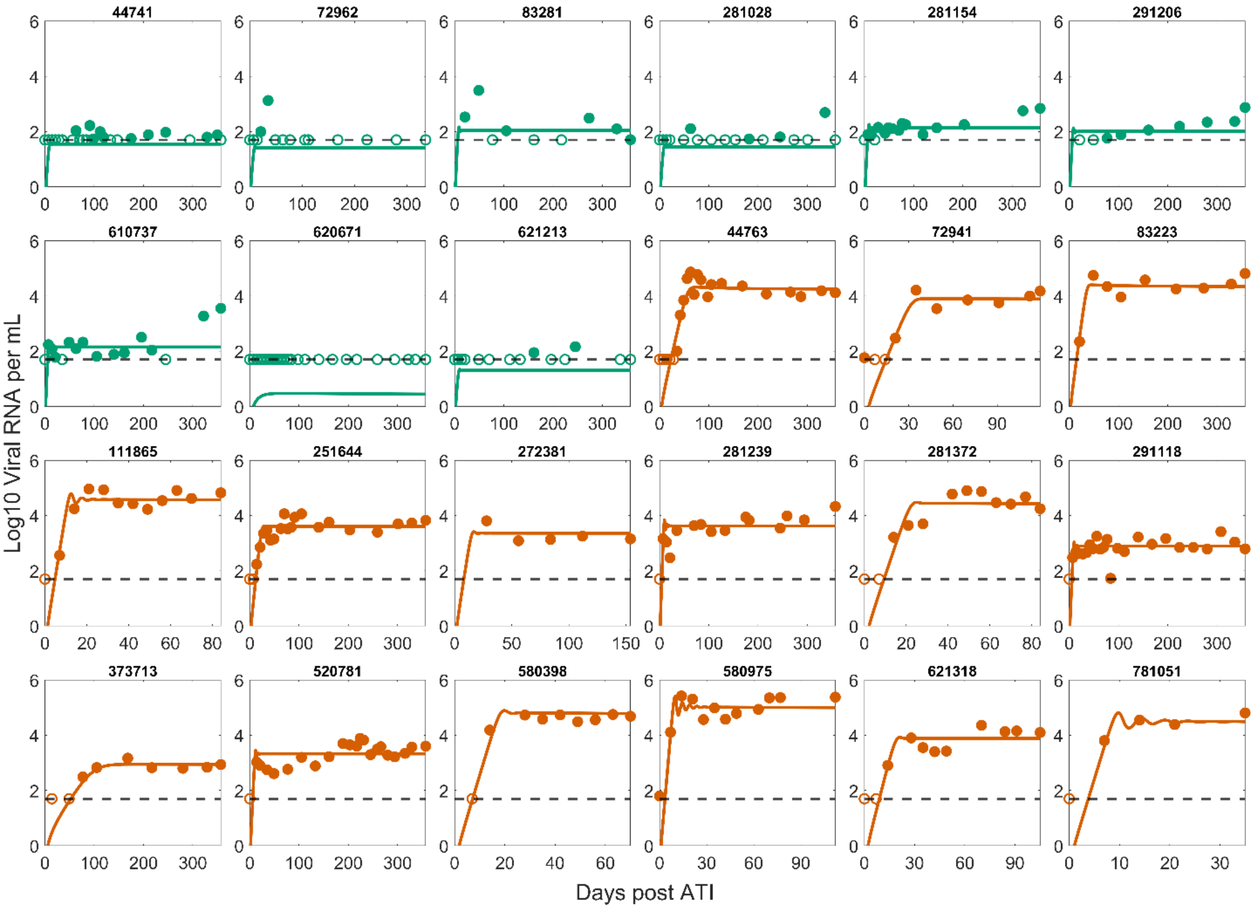

Figure S2. Best fit of Simplified Model 1 with a covariate on K_B to the post-ATI data from Sharaf et al. [57]. Lines show the model predictions using the best fit parameter values from Simplified Model 1 with a covariate on K_B. Green indicates PTC. Dark orange indicates NC. The horizontal dashed line is the limit of detection (50 viral RNA copies/mL). Open circles are data points below the limit of detection. Filled circles are data points above the limit of detection.

bioRxiv preprint doi: https://doi.org/10.1101/2024.05.03.592318; this version posted May 5, 2024. The copyright holder for this preprint (which was not certified by peer review) is the author/funder. This article is a US Government work. It is not subject to copyright under 17 USC 105 and is also made available for use under a CC0 license.

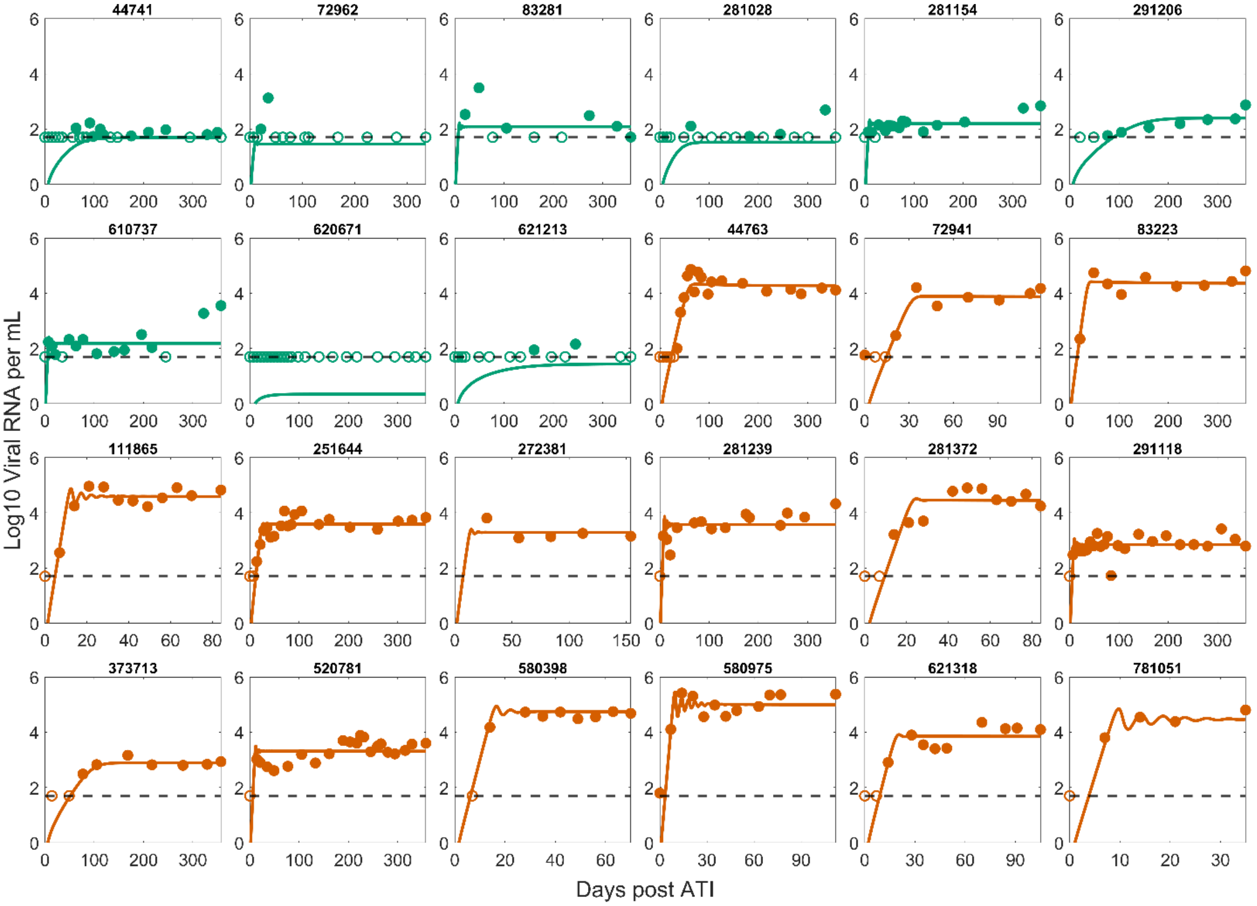

Figure S3. Best fit of the Simplified Model 2 to the post-ATI data from Sharaf et al. [57]. Green indicates PTC. Dark orange indicates NC. The horizontal dashed line is the limit of detection. Open circles are data points below the limit of detection (50 viral RNA copies/mL). Filled circles are data points above the limit of detection.

bioRxiv preprint doi: https://doi.org/10.1101/2024.05.03.592318; this version posted May 5, 2024. The copyright holder for this preprint (which was not certified by peer review) is the author/funder. This article is a US Government work. It is not subject to copyright under 17 USC 105 and is also made available for use under a CC0 license.

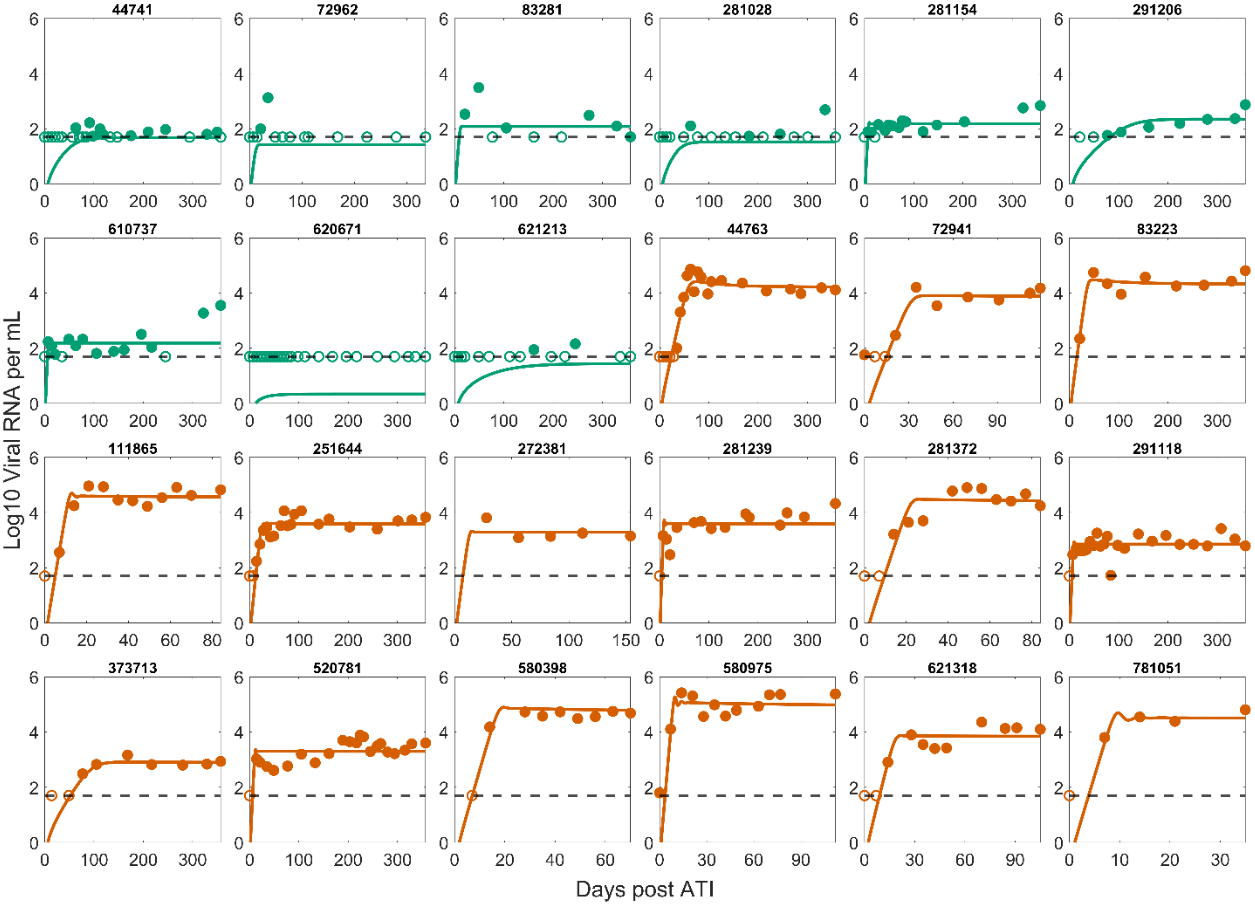

Figure S4. Best fit of the Simplified Model 3 to the post-ATI data from Sharaf et al. [57]. Green indicates PTC. Dark orange indicates NC. The horizontal dashed line is the limit of detection. Open circles are data points below the limit of detection (50 viral RNA copies/mL). Filled circles are data points above the limit of detection.

bioRxiv preprint doi: https://doi.org/10.1101/2024.05.03.592318; this version posted May 5, 2024. The copyright holder for this preprint (which was not certified by peer review) is the author/funder. This article is a US Government work. It is not subject to copyright under 17 USC 105 and is also made available for use under a CC0 license.

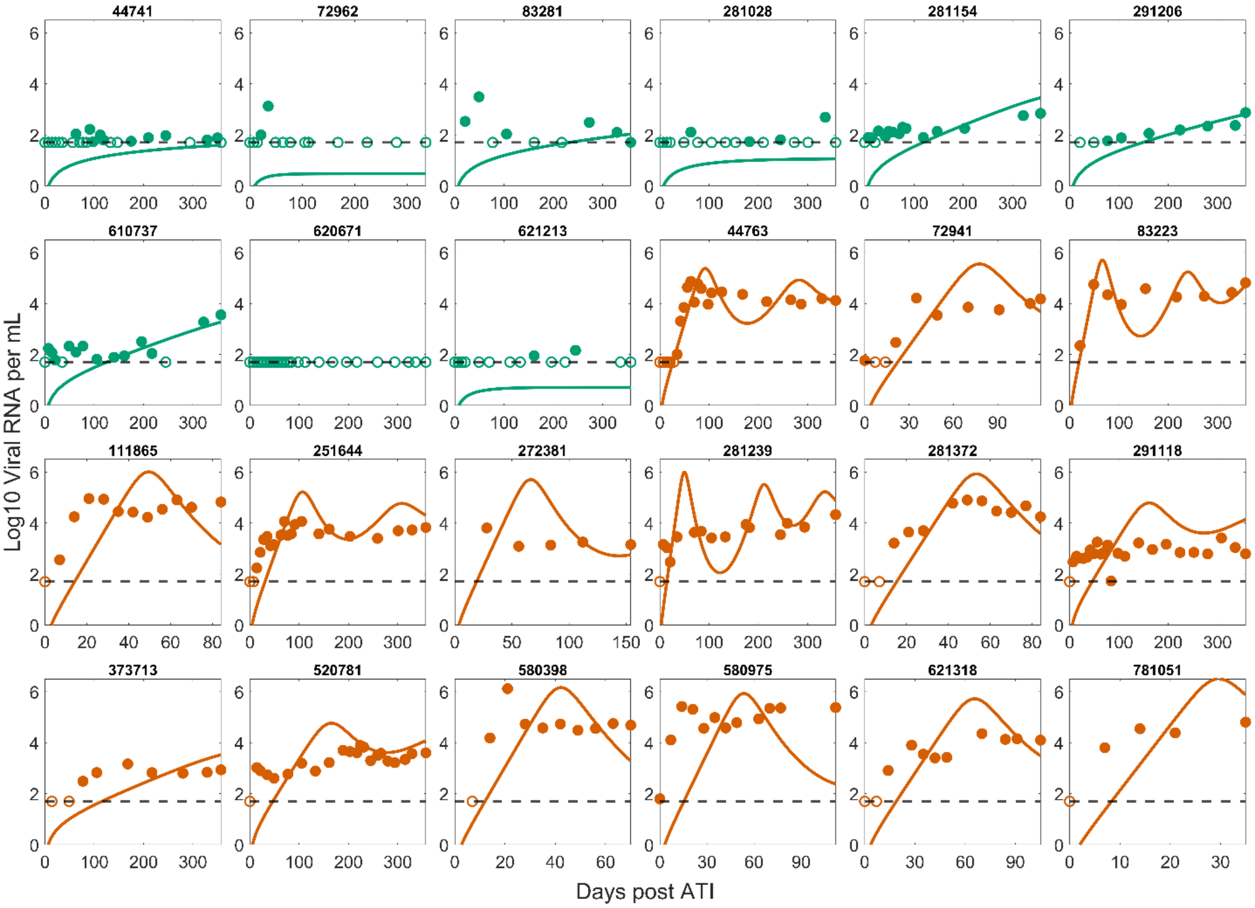

Figure S5. Best fit of the Simplified Model 4 to the post-ATI data from Sharaf et al. [57]. Green indicates PTC. Dark orange indicates NC. The horizontal dashed line is the limit of detection. Open circles are data points below the limit of detection (50 viral RNA copies/mL). Filled circles are data points above the limit of detection.

bioRxiv preprint doi: https://doi.org/10.1101/2024.05.03.592318; this version posted May 5, 2024. The copyright holder for this preprint (which was not certified by peer review) is the author/funder. This article is a US Government work. It is not subject to copyright under 17 USC 105 and is also made available for use under a CC0 license.

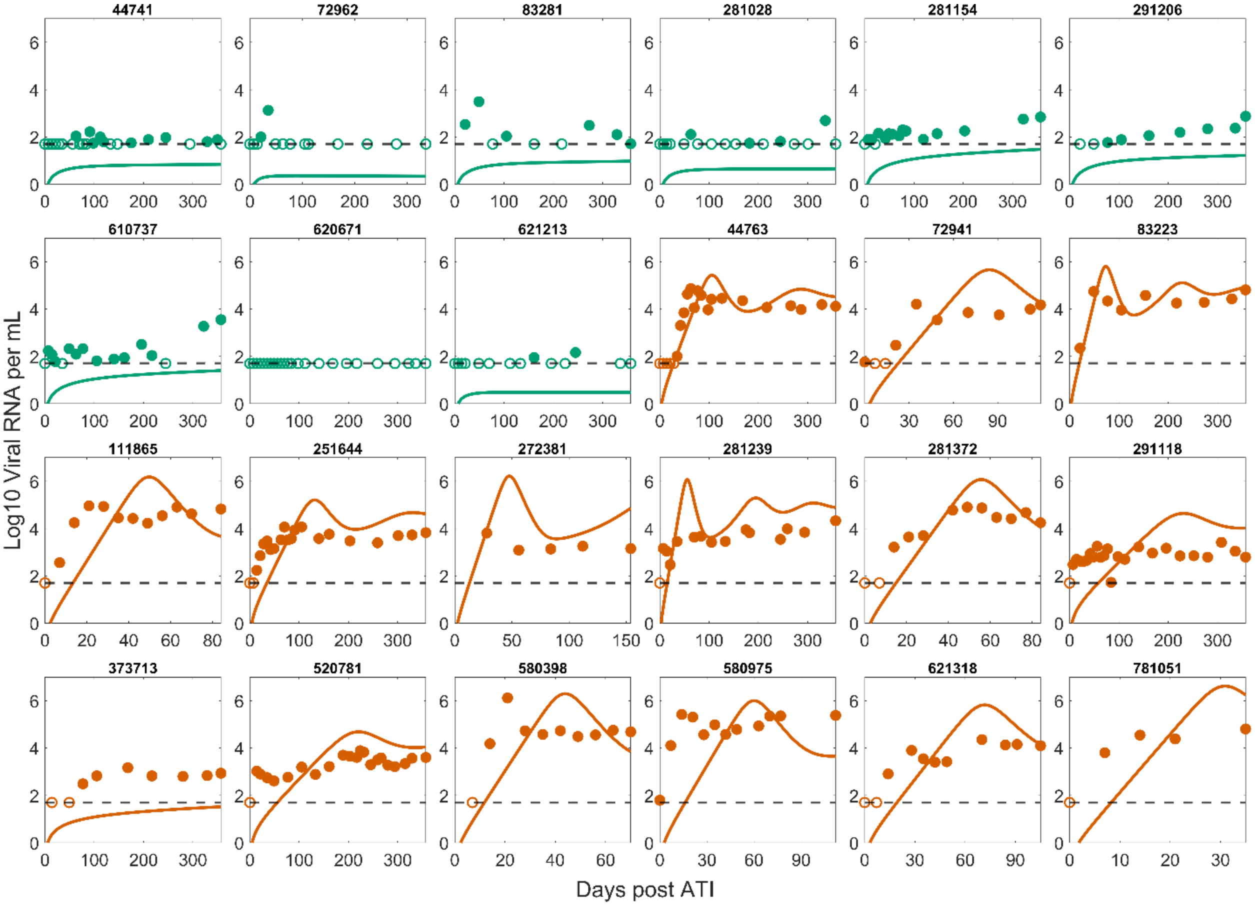

Figure S6. Best fit of the Simplified Model 5 to the post-ATI data from Sharaf et al. [57]. Green indicates PTC. Dark orange indicates NC. The horizontal dashed line is the limit of detection. Open circles are data points below the limit of detection (50 viral RNA copies/mL). Filled circles are data points above the limit of detection.

bioRxiv preprint doi: https://doi.org/10.1101/2024.05.03.592318; this version posted May 5, 2024. The copyright holder for this preprint (which was not certified by peer review) is the author/funder. This article is a US Government work. It is not subject to copyright under 17 USC 105 and is also made available for use under a CC0 license.

S3. Stratification of best fit parameters

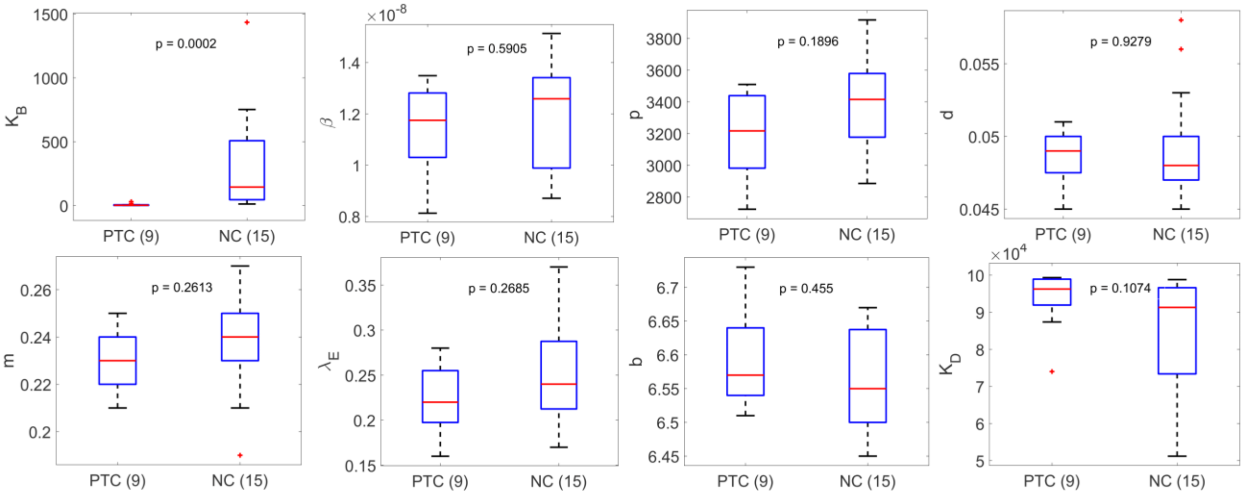

Figure S7. Summary of best-fit parameters in the Conway & Perelson Model stratified based on PTC or NC. The difference in the values of K_B is the most significant between PTC and NC.

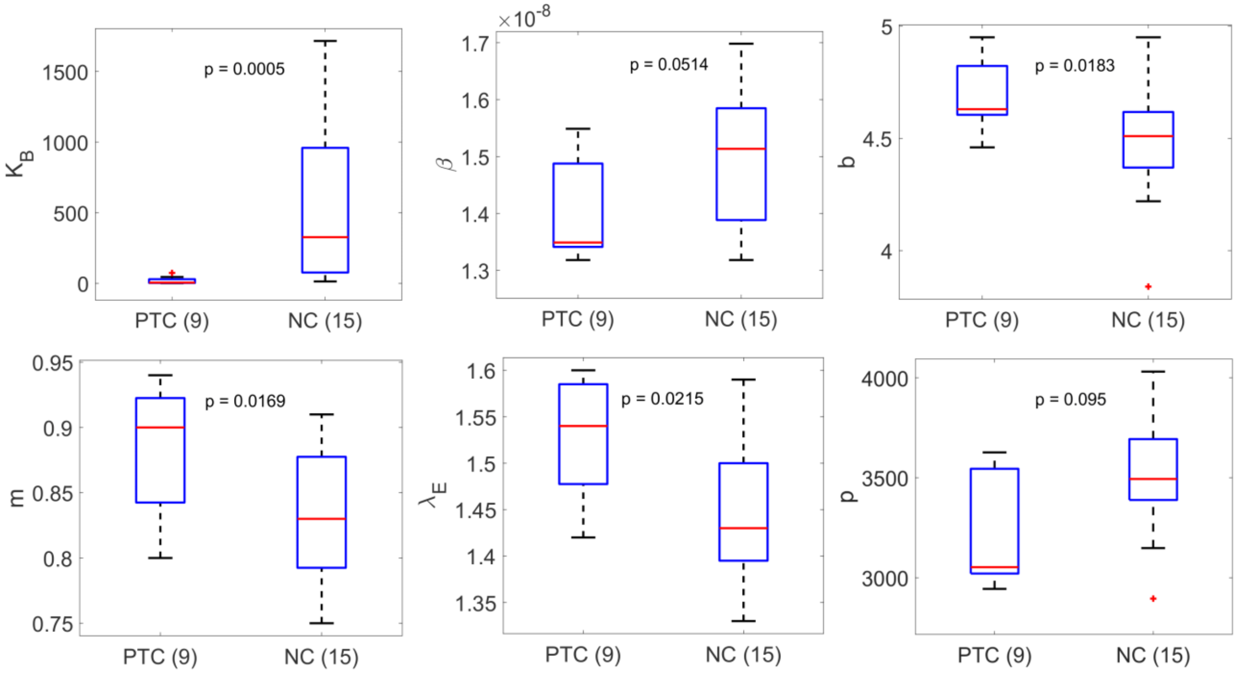

Figure S8. Summary of best fit parameters in Simplified Model 1 stratified based on PTC or NC. The difference in the values of K_B is the most significant between PTC and NC.

bioRxiv preprint doi: https://doi.org/10.1101/2024.05.03.592318; this version posted May 5, 2024. The copyright holder for this preprint (which was not certified by peer review) is the author/funder. This article is a US Government work. It is not subject to copyright under 17 USC 105 and is also made available for use under a CC0 license.

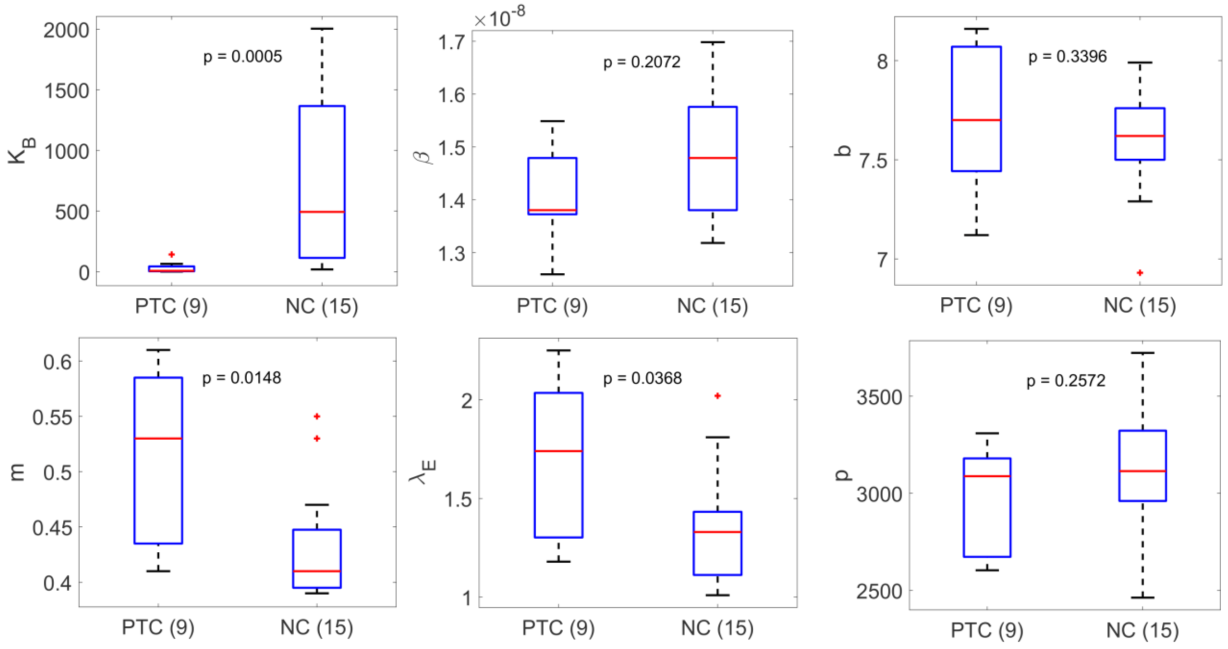

Figure S9. Summary of best-fit parameters in the Simplified Model 2 stratified based on PTC or NC. The difference in the values of K_B is the most significant between PTC and NC.

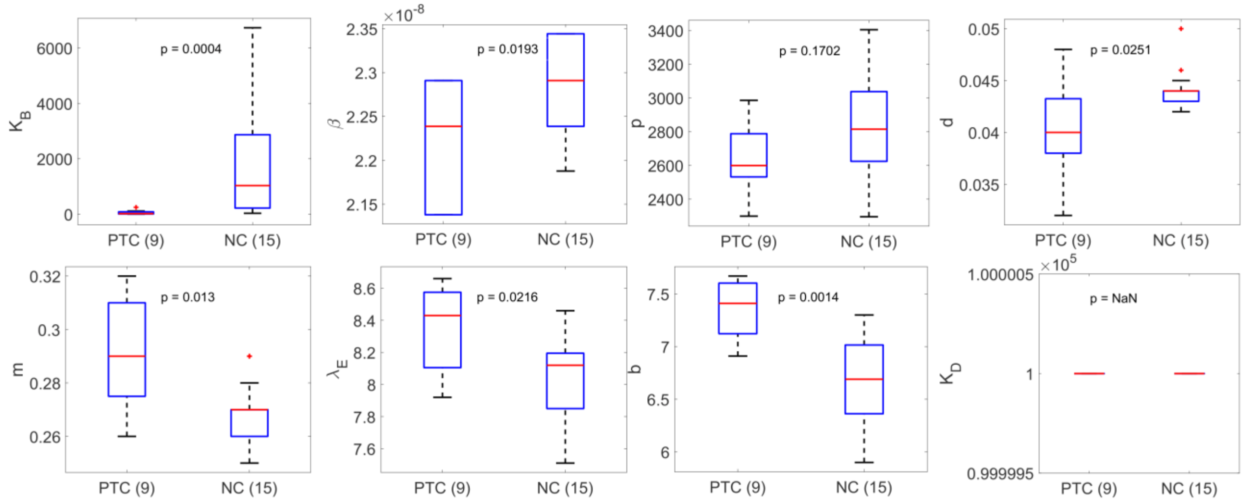

Figure S10. Summary of best-fit parameters in the Simplified Model 3 stratified based on PTC or NC. The difference in the values of K_B is the most significant between PTC and NC.

bioRxiv preprint doi: https://doi.org/10.1101/2024.05.03.592318; this version posted May 5, 2024. The copyright holder for this preprint (which was not certified by peer review) is the author/funder. This article is a US Government work. It is not subject to copyright under 17 USC 105 and is also made available for use under a CC0 license.

S4. Best fit parameters for each individual

Parameter	*Best fit values (population estimate)*	*Reference values*
β	1.20×10^{-8} $mL\ (HIV\ RNA\ copies)^{-1} day^{-1}$	1.5×10^{-8} $mL\ (HIV\ RNA\ copies)^{-1} day^{-1}$
λ_E	$0.25\ cells\ mL^{-1} day^{-1}$	$1\ cells\ mL^{-1} day^{-1}$
m	$0.24\ mL\ cells^{-1} day^{-1}$	$0.42\ mL\ cells^{-1} day^{-1}$
b	$6.57\ day^{-1}$	$1\ day^{-1}$
K_B	$29.35\ cells\ mL^{-1}$	$0.1\ cells\ mL^{-1}$
p	$3337\ (HIV\ RNA\ copies)\ day^{-1}$	$2000\ (HIV\ RNA\ copies)\ day^{-1}$
K_D	$95188\ cells\ mL^{-1}$	$5\ cells\ mL^{-1}$
d	$0.05\ day^{-1}$	$2\ day^{-1}$

Table S2. Best-fit population parameters for the Conway & Perelson Model vs. reference values from Conway and Perelson [49]. Note the estimated values of K_D and d result in a negligible effect of exhaustion.

Parameter	*Best fit values (population estimate)*	*Reference values*
β	1.51×10^{-8} $mL(HIV\ RNA\ copies)^{-1}\ day^{-1}$	1.5×10^{-8} $mL\ (HIV\ RNA\ copies)^{-1} day^{-1}$
λ_E	$1.33\ cells\ mL^{-1} day^{-1}$	$1\ cells\ mL^{-1} day^{-1}$
m	$0.44\ mL\ cells^{-1} day^{-1}$	$0.42\ mL\ cells^{-1} day^{-1}$
b	$7.66\ day^{-1}$	$1\ day^{-1}$
K_B	$65.99\ cells\ mL^{-1}$	$0.1\ cells\ mL^{-1}$
p	$3104\ (HIV\ RNA\ copies)\ day^{-1}$	$2000\ (HIV\ RNA\ copies)\ day^{-1}$

Table S3. Best-fit population parameters for the Simplified Model 2 vs. reference values from Conway and Perelson [49].

Parameter	*Best fit values (population estimate)*	*Reference values*
β	2.29×10^{-8} $mL\ (HIV\ RNA\ copies)^{-1} day^{-1}$	1.5×10^{-8} $mL\ (HIV\ RNA\ copies)^{-1} day^{-1}$
λ_E	$8.06\ cells\ mL^{-1} day^{-1}$	$1\ cells\ mL^{-1} day^{-1}$
m	$0.27\ mL\ cells^{-1} day^{-1}$	$0.42\ mL\ cells^{-1} day^{-1}$
b	$6.8\ day^{-1}$	$1\ day^{-1}$
K_B	$122.97\ cells\ mL^{-1}$	$0.1\ cells\ mL^{-1}$
p	$2798\ (HIV\ RNA\ copies)\ day^{-1}$	$2000\ (HIV\ RNA\ copies)\ day^{-1}$
K_D	$100000\ cells\ mL^{-1}$	$5\ cells\ mL^{-1}$
d	$0.044\ day^{-1}$	$2\ day^{-1}$

Table S4. Best-fit population parameters for the Simplified Model 3 vs. reference values from Conway and Perelson [49]. Note that the estimated values of K_D and d together imply the exhaustion effect is very small, which may be because of this particular set of participants or that it cannot be observed from the limited data.

bioRxiv preprint doi: https://doi.org/10.1101/2024.05.03.592318; this version posted May 5, 2024. The copyright holder for this preprint (which was not certified by peer review) is the author/funder. This article is a US Government work. It is not subject to copyright under 17 USC 105 and is also made available for use under a CC0 license.

Parameter	*Best fit values (population estimate)*	*Reference values*
β	2.04×10^{-8} $mL\ (HIV\ RNA\ copies)^{-1} day^{-1}$	1.5×10^{-8} $mL\ (HIV\ RNA\ copies)^{-1} day^{-1}$
m^*	$1.4\ mL\ day^{-1}$	
p	$2855\ (HIV\ RNA\ copies)\ day^{-1}$	$2000\ (HIV\ RNA\ copies)\ day^{-1}$

Table S5. Best-fit population parameters for the Simplified Model 4 vs. reference values from Conway and Perelson [49]. Note that m^* is not the same as m.

Parameter	*Best fit values (population estimate)*	*Reference values*
β	1.48×10^{-8} $mL\ (HIV\ RNA\ copies)^{-1} day^{-1}$	1.5×10^{-8} $mL\ (HIV\ RNA\ copies)^{-1} day^{-1}$
m^*	$1.18\ mL\ day^{-1}$	
p	$3608\ (HIV\ RNA\ copies)\ day^{-1}$	$2000\ (HIV\ RNA\ copies)\ day^{-1}$

Table S6. Best-fit population parameters for the Simplified Model 5 vs. reference values from Conway and Perelson [49]. Note that m^* is not the same as m.

bioRxiv preprint doi: https://doi.org/10.1101/2024.05.03.592318; this version posted May 5, 2024. The copyright holder for this preprint (which was not certified by peer review) is the author/funder. This article is a US Government work. It is not subject to copyright under 17 USC 105 and is also made available for use under a CC0 license.

ID	$-log_{10}(\beta)$	λ_E	b	K_B	m	d	K_D	p
44741	7.9	0.21	6.51	0.77	0.22	0.046	74015	2935
44763	8.06	0.23	6.49	410.9	0.24	0.058	96410	3136
72941	8.05	0.23	6.5	131.1	0.23	0.05	91311	3416
72962	7.87	0.19	6.61	0.41	0.21	0.049	96258	3510
83223	8.01	0.22	6.55	470.7	0.23	0.05	71299	3095
83281	7.87	0.16	6.57	1.86	0.22	0.048	98839	3427
111865	7.85	0.37	6.67	680.2	0.26	0.048	93935	3592
251644	7.99	0.25	6.64	72.74	0.24	0.046	98802	3168
272381	7.87	0.24	6.5	26.73	0.23	0.047	97931	3461
281028	7.93	0.22	6.73	0.6	0.24	0.049	99161	3098
281154	7.93	0.2	6.55	3	0.22	0.045	87383	3334
281239	7.9	0.17	6.53	73.98	0.19	0.045	98378	3659
281372	7.97	0.17	6.52	520.2	0.21	0.05	71515	3208
291118	7.89	0.32	6.64	11.83	0.26	0.047	92294	3542
291206	8.09	0.27	6.51	13.46	0.24	0.048	97834	2998
373713	8.06	0.28	6.48	27.61	0.25	0.048	90667	2886
520781	7.88	0.2	6.67	36.46	0.22	0.047	88941	3423
580398	7.88	0.29	6.45	752.7	0.27	0.053	96695	3328
580975	7.82	0.32	6.61	1436	0.24	0.045	68560	3917
610737	7.9	0.25	6.59	2.41	0.23	0.05	93462	3478
620671	8.08	0.28	6.57	29.73	0.25	0.05	95571	2723
621213	7.96	0.25	6.73	0.41	0.24	0.051	99333	3217
621318	7.94	0.21	6.63	144.5	0.24	0.05	79011	3286
781051	7.82	0.28	6.61	317.3	0.25	0.056	51197	3670
Mean	7.94	0.24	6.58	215.2	0.23	0.049	88700	3313
SD (%)	1.02	21.13	1.16	158.5	7.58	6.43	14.02	8.28

Table S7. Individual best-fit parameters – Conway & Perelson model.

bioRxiv preprint doi: https://doi.org/10.1101/2024.05.03.592318; this version posted May 5, 2024. The copyright holder for this preprint (which was not certified by peer review) is the author/funder. This article is a US Government work. It is not subject to copyright under 17 USC 105 and is also made available for use under a CC0 license.

ID	$-log_{10}(\beta)$	λ_E	b	K_B	m	p
44741	7.87	1.6	4.63	14.67	0.92	3053
44763	7.85	1.43	4.67	1716	0.87	2896
72941	7.87	1.55	4.22	326.9	0.89	3422
72962	7.85	1.49	4.95	0.99	0.9	3468
83223	7.86	1.51	4.23	1222	0.88	3225
83281	7.83	1.49	4.78	2.95	0.85	3536
111865	7.8	1.38	4.43	568.4	0.78	3707
251644	7.86	1.54	4.63	149	0.89	3463
272381	7.82	1.46	4.58	42.88	0.83	3494
281028	7.87	1.58	4.61	5.08	0.91	3053
281154	7.82	1.44	4.95	3.33	0.8	3570
281239	7.79	1.39	4.35	52.61	0.79	3787
281372	7.84	1.47	4.43	795.3	0.84	3379
291118	7.8	1.41	4.95	13.41	0.8	3651
291206	7.88	1.56	4.46	73.62	0.89	3040
373713	7.88	1.59	4.64	161.2	0.91	3148
520781	7.81	1.41	4.51	34.97	0.81	3650
580398	7.82	1.43	4.52	1422	0.81	3494
580975	7.77	1.33	3.84	1014	0.75	4031
610737	7.81	1.42	4.68	2.92	0.82	3627
620671	7.88	1.6	4.59	45.02	0.94	2964
621213	7.87	1.54	4.62	24.08	0.93	2945
621318	7.84	1.47	4.51	191.5	0.84	3418
781051	7.77	1.34	4.52	411.5	0.75	3881
Mean	7.84	1.48	4.55	345.6	0.85	3413
SD (%)	0.43	5.37	5.29	144.1	6.51	8.95

Table S8. Individual best-fit parameters – Simplified Model 1.

bioRxiv preprint doi: https://doi.org/10.1101/2024.05.03.592318; this version posted May 5, 2024. The copyright holder for this preprint (which was not certified by peer review) is the author/funder. This article is a US Government work. It is not subject to copyright under 17 USC 105 and is also made available for use under a CC0 license.

ID	$-log_{10}(\beta)$	λ_E	b	K_B	m	p	covariate
44741	7.82	0.54	5.23	0.94	1.69	3097	1
44763	7.85	0.52	5.13	1488	1.72	2657	2
72941	7.87	0.6	5.15	359.8	1.76	3123	2
72962	7.83	0.54	5.75	0.78	1.69	3179	1
83223	7.86	0.55	4.25	878.9	1.72	2977	2
83281	7.8	0.48	5.09	2.17	1.69	3261	1
111865	7.78	0.45	4.97	603.1	1.65	3424	2
251644	7.85	0.58	5.35	160.7	1.74	3062	2
272381	7.81	0.54	6.18	75.81	1.71	3104	2
281028	7.82	0.54	5.14	0.74	1.66	3112	1
281154	7.8	0.48	5.29	2.89	1.7	3253	1
281239	7.77	0.47	5.78	80.96	1.66	3466	2
281372	7.83	0.52	4.61	699.7	1.73	3066	2
291118	7.78	0.51	6.41	20.21	1.75	3331	2
291206	7.8	0.49	4.85	1.86	1.69	3276	1
373713	7.86	0.63	5.26	201.7	1.92	2764	2
520781	7.79	0.51	6.24	55.49	1.7	3241	2
580398	7.82	0.48	4.2	1104	1.7	3179	2
580975	7.76	0.42	4.45	1125	1.6	3749	2
610737	7.79	0.47	5.38	2.74	1.68	3351	1
620671	7.86	0.66	5.49	0.92	1.86	2722	1
621213	7.82	0.55	5.67	0.64	1.71	3078	1
621318	7.83	0.55	5.41	227.7	1.67	3106	2
781051	7.76	0.43	5.24	455.6	1.63	3613	2
Mean	7.82	0.52	5.27	1.52/502.4	1.71	3175	
SD (%)	0.41	11.07	10.57	56.04/89.14	3.84	7.85	

821 Table S9. Individual best-fit parameters – Simplified Model 1 with a covariate on K_B (1 is PTC
822 and 2 is NC). For K_B, the mean and SD (%) are reported for individual group PTC/NC.

823

bioRxiv preprint doi: https://doi.org/10.1101/2024.05.03.592318; this version posted May 5, 2024. The copyright holder for this preprint (which was not certified by peer review) is the author/funder. This article is a US Government work. It is not subject to copyright under 17 USC 105 and is also made available for use under a CC0 license.

ID	$-log_{10}(\beta)$	λ_E	b	K_B	m	p
44741	7.86	1.89	8.06	21.76	0.6	2681
44763	7.86	1.41	7.99	1950	0.41	2462
72941	7.88	1.81	7.48	493.7	0.53	3127
72962	7.85	1.55	8.16	1.24	0.47	3088
83223	7.86	1.52	6.93	1439	0.47	2788
83281	7.83	1.31	7.94	3.81	0.42	3177
111865	7.8	1.1	7.62	971.2	0.39	3341
251644	7.88	1.33	7.78	179.1	0.44	2955
272381	7.83	1.34	7.66	64.83	0.44	3085
281028	7.86	1.74	7.15	7.44	0.54	2603
281154	7.83	1.28	8.1	5.15	0.44	3187
281239	7.79	1.15	7.81	92.8	0.39	3406
281372	7.85	1.44	7.56	1147	0.44	2975
291118	7.81	1.21	7.81	19.49	0.41	3267
291206	7.86	1.99	7.54	142.6	0.53	2648
373713	7.87	2.02	7.58	235.1	0.55	2762
520781	7.82	1.2	7.7	58.35	0.41	3220
580398	7.82	1.01	7.58	1586	0.41	3114
580975	7.77	1.04	7.29	2004	0.39	3723
610737	7.81	1.18	7.7	4.1	0.41	3309
620671	7.87	2.17	7.66	65.31	0.58	2694
621213	7.9	2.25	7.12	37.28	0.61	3122
621318	7.85	1.41	7.47	266.1	0.45	3046
781051	7.77	1.08	7.67	655.7	0.39	3561
Mean	7.84	1.48	7.64	477.1	0.46	3056
SD (%)	0.43	24.59	3.94	135.8	15.01	10.03

Table S10. Individual best-fit parameters – Simplified Model 2.

bioRxiv preprint doi: https://doi.org/10.1101/2024.05.03.592318; this version posted May 5, 2024. The copyright holder for this preprint (which was not certified by peer review) is the author/funder. This article is a US Government work. It is not subject to copyright under 17 USC 105 and is also made available for use under a CC0 license.

ID	$-log_{10}(\beta)$	λ_E	b	K_B	m	d	K_D	p
44741	7.66	8.54	7.61	40.57	0.31	0.042	100000	2542
44763	7.64	8.12	7.1	6727	0.26	0.05	100000	2296
72941	7.65	8.37	7.3	1230	0.29	0.043	100000	2624
72962	7.65	8.62	7.67	3.52	0.28	0.038	100000	2665
83223	7.65	8.21	6.35	4088	0.27	0.046	100000	2500
83281	7.64	8.22	7.22	10.19	0.28	0.04	100000	2744
111865	7.63	7.8	6.42	1586	0.26	0.044	100000	3049
251644	7.65	8.25	6.2	398.3	0.28	0.044	100000	2625
272381	7.64	8.05	6.8	122.4	0.27	0.044	100000	2815
281028	7.67	8.66	7.58	19.98	0.31	0.038	100000	2600
281154	7.64	8.14	7.6	9.38	0.26	0.04	100000	2920
281239	7.63	7.83	7.18	161.7	0.25	0.045	100000	3148
281372	7.64	8.15	6.24	2252	0.27	0.043	100000	2698
291118	7.63	7.91	6.97	33.62	0.26	0.043	100000	3006
291206	7.67	8	6.91	246.8	0.31	0.048	100000	2504
373713	7.66	8.46	7.03	542.3	0.29	0.042	100000	2436
520781	7.64	8	6.94	106.0	0.26	0.042	100000	2945
580398	7.64	8.12	6.4	4174	0.27	0.042	100000	2859
580975	7.63	7.6	5.9	3074	0.25	0.043	100000	3405
610737	7.64	7.92	7.41	8.13	0.26	0.044	100000	2986
620671	7.65	8.43	6.95	112.5	0.29	0.043	100000	2299
621213	7.67	8.56	7.18	75	0.32	0.032	100000	2582
621318	7.65	8.13	6.69	548.6	0.27	0.044	100000	2733
781051	7.63	7.51	6.5	1034	0.25	0.044	100000	3259
Mean	7.65	8.15	6.92	1109	0.28	0.043	100000	2760
SD (%)	0.16	3.67	7.04	154.5	7.32	8.05	0	10.20

Table S11. Individual best-fit parameters – Simplified Model 3.

bioRxiv preprint doi: https://doi.org/10.1101/2024.05.03.592318; this version posted May 5, 2024. The copyright holder for this preprint (which was not certified by peer review) is the author/funder. This article is a US Government work. It is not subject to copyright under 17 USC 105 and is also made available for use under a CC0 license.

ID	$-log_{10}(\beta)$	m	p
44741	7.69	1.44	2767
44763	7.69	1.38	2855
72941	7.68	1.38	2886
72962	7.69	1.46	2744
83223	7.68	1.36	2901
83281	7.69	1.45	2775
111865	7.68	1.33	2962
251644	7.68	1.41	2853
272381	7.68	1.37	2911
281028	7.69	1.45	2759
281154	7.69	1.45	2790
281239	7.68	1.32	2951
281372	7.68	1.35	2958
291118	7.69	1.44	2836
291206	7.69	1.44	2775
373713	7.69	1.44	2782
520781	7.69	1.44	2841
580398	7.68	1.32	3020
580975	7.68	1.34	2958
610737	7.69	1.44	2787
620671	7.69	1.5	2669
621213	7.69	1.45	2751
621318	7.68	1.37	2914
781051	7.68	1.27	3148
Mean	7.69	1.4	2858
SD (%)	0.065	4.08	3.69

Table S12. Individual best-fit parameters – Simplified Model 4.

bioRxiv preprint doi: https://doi.org/10.1101/2024.05.03.592318; this version posted May 5, 2024. The copyright holder for this preprint (which was not certified by peer review) is the author/funder. This article is a US Government work. It is not subject to copyright under 17 USC 105 and is also made available for use under a CC0 license.

ID	$-log_{10}(\beta)$	m	p
44741	7.84	1.21	3519
44763	7.83	1.17	3607
72941	7.83	1.17	3643
72962	7.84	1.22	3496
83223	7.83	1.14	3643
83281	7.84	1.21	3531
111865	7.82	1.13	3725
251644	7.83	1.2	3612
272381	7.81	1.14	3745
281028	7.84	1.21	3512
281154	7.84	1.21	3547
281239	7.82	1.14	3687
281372	7.82	1.14	3699
291118	7.83	1.21	3554
291206	7.84	1.21	3529
373713	7.84	1.21	3536
520781	7.83	1.21	3571
580398	7.81	1.13	3762
580975	7.82	1.15	3706
610737	7.84	1.21	3544
620671	7.85	1.24	3414
621213	7.84	1.22	3502
621318	7.82	1.16	3664
781051	7.8	1.1	3861
Mean	7.83	1.18	3609
SD (%)	0.15	3.21	2.87

Table S13. Individual best-fit parameters – Simplified Model 5.

bioRxiv preprint doi: https://doi.org/10.1101/2024.05.03.592318; this version posted May 5, 2024. The copyright holder for this preprint (which was not certified by peer review) is the author/funder. This article is a US Government work. It is not subject to copyright under 17 USC 105 and is also made available for use under a CC0 license.

S5. Analytical approximation of the early viral rebound dynamics

In order to better explain why K_B separates the NC and PTC participants, we approximate analytically the viral set point after rebound for Simplified Model 1:

$$\frac{dT}{dt} = \lambda_T - d_T T - \beta V T$$

$$\frac{dI}{dt} = (1 - f_L)\beta V T + aL - \delta I - mEI$$

$$\frac{dL}{dt} = f_L \beta V T - d_L L - aL + \rho L$$

$$\frac{dE}{dt} = \lambda_E + \frac{bEI}{K_B + I} - d_E E$$

$$V = \frac{p}{c} I.$$

We are interested in the viral set point attained < 1 year post-ATI and as HIV-1 infection progresses slowly, we approximate the target cell population by its disease-free equilibrium $T = \lambda_T / d_T$. It is worth pointing out that PWH, who experienced rapid viral rebound and potentially rapid decline of target cells, re-started ART. This approximation also works for the period before ART.

In this deterministic model, the contribution from latent cells to the viral rebound dynamics (e.g., the rate that virus becomes detectable and the magnitude of the set point viral load) is negligible. Thus, we assume that $L = 0$ for this approximation, which will simplify the algebraic details further. Together, these considerations result in the following transient system which approximates the early viral rebound dynamics of the full model:

$$I' = \left((1 - f_L)\beta \left(\frac{p}{c}\right) \left(\frac{\lambda_T}{d_T}\right) - \delta - mE \right) I$$

$$E' = \lambda_E + \frac{bEI}{K_B + I} - d_E E.$$

This approximated system with one infected cell compartment and one effector cell compartment is inspired by a previous study (Baral et al., 2019). Now, solving for $E' = 0$ gives

$$E_{ss} = \frac{\lambda_E (K_B + I)}{d_E (K_B + I) - bI}.$$

Substituting this expression for E into the I' equation,

$$I' = \left((1 - f_L)\beta \left(\frac{p}{c}\right) \left(\frac{\lambda_T}{d_T}\right) - \delta - m \frac{\lambda_E (K_B + I)}{d_E (K_B + I) - bI} \right) I.$$

Setting $I' = 0$ and solving for the non-zero steady state I_{ss} gives

bioRxiv preprint doi: https://doi.org/10.1101/2024.05.03.592318; this version posted May 5, 2024. The copyright holder for this preprint (which was not certified by peer review) is the author/funder. This article is a US Government work. It is not subject to copyright under 17 USC 105 and is also made available for use under a CC0 license.

$$I_{ss} = \frac{\left[m\lambda_E - \left((1-f_L)\beta\left(\frac{p}{c}\right)\left(\frac{\lambda_T}{d_T}\right) - \delta\right)d_E\right]K_B}{\left((1-f_L)\beta\left(\frac{p}{c}\right)\left(\frac{\lambda_T}{d_T}\right) - \delta\right)(d_E - b) - m\lambda_E}.$$

The corresponding critical value V_c for $V(t)$ is given by

$$V_{ss} = \frac{p}{c}I_{ss} = \frac{p}{c}\frac{\left[m\lambda_E - \left((1-f_L)\beta\left(\frac{p}{c}\right)\left(\frac{\lambda_T}{d_T}\right) - \delta\right)d_E\right]K_B}{\left((1-f_L)\beta\left(\frac{p}{c}\right)\left(\frac{\lambda_T}{d_T}\right) - \delta\right)(d_E - b) - m\lambda_E}.$$

Figure S11 shows a comparison of V_{ss} with the predicted viral load set-point obtained with the full model dynamics.

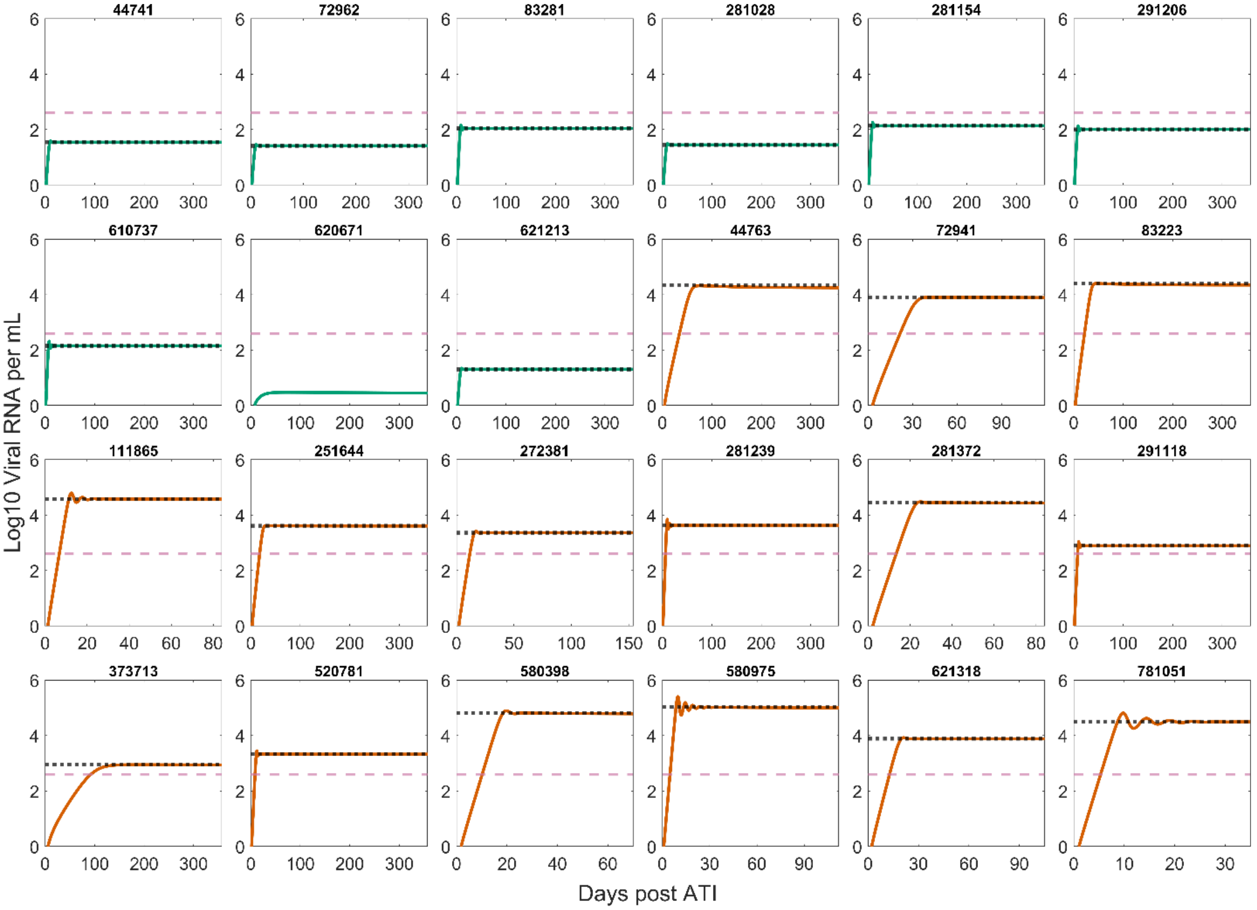

Figure S11. Approximation of the set point viral load V_{ss} using the best fit parameters for each participant compared with the numerical solution of Simplified Model 1 (Eqn. 2). The horizontal black dotted line is the approximation V_{ss} (Eqn. 3). Green and dark orange curves correspond to the viral load of the PTC and NC participants, respectively, as predicted by the Simplified Model

bioRxiv preprint doi: https://doi.org/10.1101/2024.05.03.592318; this version posted May 5, 2024. The copyright holder for this preprint (which was not certified by peer review) is the author/funder. This article is a US Government work. It is not subject to copyright under 17 USC 105 and is also made available for use under a CC0 license.

1. The horizontal pink dashed line is the 400 viral RNA copies/mL threshold used in the classification of PTC. For participant number 620671, V_{ss} is at 0.61 viral RNA copies/mL, which is the full model predicted viral set point after day ~12000.

To arrive at this approximation of the viral set point, we made several crude simplifications, where the assumptions of limited exhaustion $\left(\frac{dEI}{K_D+I} \approx 0\right)$ and constant target cells $\left(T = \frac{\lambda_T}{d_T}\right)$ resulted in the removal of one of the two non-extinction steady states of the full model. However, it is not clear whether or when both steady states are positive since the algebraic calculation is fairly complicated for the full model. The model also contains a disease-free steady state, which is unstable assuming the baseline immune response is not sufficient to clear the virus on its own.
